# Feasibility of the Manual Diaphragm Release Technique in Neurocritical Patients on Mechanical Ventilation: A Pilot Randomized Controlled Trial

**DOI:** 10.3390/medsci14010001

**Published:** 2025-12-19

**Authors:** Elis Fernanda Araújo Lima de Oliveira, Helga Cecília Muniz de Souza, Heitor Fernandes Silveira Cavalini, Fabianne Maisa de Novaes Assis Dantas, Victor Ribeiro Neves, Fernando de Aguiar Lemos, Marcelo Gama de Abreu, Paulo André Freire Magalhães

**Affiliations:** 1 Graduate Program in Rehabilitation and Functional Performance (PPGRDF), Universidade de Pernambuco (UPE), Petrolina 56328-900, Brazil; elisfernanda89@gmail.com (E.F.A.L.d.O.); heitor.cavalini@upe.br (H.F.S.C.); fabianne.assis@upe.br (F.M.d.N.A.D.); victor.neves@upe.br (V.R.N.); 2 Hospital das Clínicas, Universidade Federal de Pernambuco (HC-UFPE), Recife 50670-901, Brazil; helgamuniz@yahoo.com.br; 3 Department of Physical Education, Universidade Federal do Vale do São Francisco (UNIVASF), Petrolina 56304-917, Brazil; fernando.aguiar@univasf.edu.br; 4 Department of Intensive Care and Resuscitation, Anesthesiology Institute, Cleveland Clinic, Cleveland, OH 44195, USA; gamadem@ccf.org

**Keywords:** diaphragm, intensive care unit, ultrasound, manual therapy

## Abstract

Introduction: This pilot randomized trial evaluated the feasibility of the Manual Diaphragm Release Technique (MDRT) in neurocritical patients on invasive mechanical ventilation (IMV) and explored its immediate effects on diaphragmatic kinematics to inform future trials. Methods: Adult neurocritical patients receiving IMV and ventilated in an assisted mode (pressure-support ventilation, PSV) at the time of enrollment were randomized to receive a single session of MDRT plus standard physiotherapy vs. a sham maneuver plus standard physiotherapy. The primary outcome was the feasibility of applying MDRT in neurocritical care patients under IMV, operationalized by the recruitment rate, protocol adherence, and incidence of intervention-related adverse events. The exploratory secondary outcomes were immediate diaphragmatic kinematics (contraction and relaxation velocities and inspiratory and expiratory excursions), which were measured by ultrasound to provide preliminary effect-size estimates for future trials. Results: Twenty neurocritical patients (10 in each group) were randomized and all completed the protocol. Baseline characteristics were comparable between groups. The study demonstrated high feasibility with 80% recruitment rate, 100% adherence, and a mean intervention time of 6.2 ± 1.1 min. No adverse events were observed during or after the intervention. Adjusted analyses revealed no detectable differences in diaphragmatic kinematics between groups after the single session. The adjusted mean differences were 0.1 mm/s (95% CI: −0.3 to 0.5; *p* = 0.50) for contraction velocity and 0.2 mm/s (95% CI: −0.05 to 0.45; *p* = 0.11) for relaxation velocity. For diaphragmatic excursion, the difference was 0.5 mm (95% CI: −1.2 to 2.2; *p* = 0.55) during inspiration and 1.0 mm (95% CI: −0.1 to 2.1; *p* = 0.08) during expiration. Conclusions: MDRT was found to be feasible for use in neurocritical patients under mechanical ventilation. Although no immediate effects on diaphragm kinematics were detected, these preliminary findings support the rationale for larger, adequately powered trials to further investigate cumulative or long-term effects. Trial registration: The trial is registered in ReBEC—Brazilian Registry of Clinical Trials under ID: RBR-3ngffwr.

## 1. Introduction

Invasive mechanical ventilation (IMV) is frequently required in neurocritical patients due to impaired consciousness, compromised airway protection, and the complex disruption of neural–respiratory integration inherent to conditions such as stroke, traumatic brain injury (TBI), and hypoxic–ischemic brain injury (HIBI). Prognosis in this group is highly heterogeneous and shaped by disease severity, secondary neurological complications, and the physiological burden of prolonged mechanical support [1]. This unique interplay between neural dysfunction and respiratory mechanics makes the preservation of diaphragmatic function especially relevant for clinical recovery in this vulnerable group.

Ventilator-induced diaphragmatic dysfunction (VIDD) is among the most pervasive complications of IMV, with an incidence ranging from 40% to 80% and measurable impairments emerging within 24–48 h of ventilation [2,3,4,5,6]. VIDD contributes to reduced inspiratory strength, delayed or failed weaning, extended ICU and hospital length of stay, and increased rehabilitation needs. Neurocritical patients appear to be at even greater risk, as sedation protocols, diminished central respiratory drive, and impaired corticospinal integration amplify the effects of mechanical unloading. In contrast to other ventilated populations (such as postoperative, medical, or trauma patients) neurocritical patients frequently exhibit reduced cortical modulation of breathing and impaired afferent feedback, conditions that may accelerate disuse atrophy and denervation processes [5,6]. This distinct neurophysiological vulnerability justifies focused feasibility research aimed at identifying interventions that preserve diaphragmatic function and may attenuate downstream disability.

The mode of ventilation further influences the trajectory of diaphragmatic integrity. Controlled ventilation markedly reduces diaphragmatic loading and activates proteolytic pathways—particularly the ubiquitin–proteasome system—alongside increased expression of atrophy-related genes such as MAFbx/atrogin-1, resulting in rapid muscle fiber atrophy and contractile dysfunction. Assisted modes such as pressure-support ventilation (PSV) partially preserve neural respiratory drive and spontaneous activity but remain insufficient to fully prevent VIDD [7]. These mechanisms collectively contribute to difficult weaning and increased healthcare resource utilization.

Despite advances in critical care, long-term preservation of respiratory muscle function remains a major challenge. Persistent respiratory muscle weakness after ICU discharge is common and can significantly impair functional recovery and quality of life. There is growing clinical emphasis on targeted interventions capable of counteracting respiratory muscle atrophy and the deleterious effects of mechanical ventilation and inactivity [8]. Interventions that can sustain diaphragmatic activity or reduce structural impediments may therefore offer clinically meaningful benefits.

Within this context, the Manual Diaphragm Release Technique (MDRT) has gained attention as a noninvasive manual approach designed to optimize diaphragmatic biomechanics. MDRT aims to reduce myofascial restriction at the costal insertions of the diaphragm, facilitate muscle relaxation, and enhance excursion and compliance through improved sliding of actin–myosin filaments and increased sarcomere extensibility [9]. Studies in healthy older adults and individuals with COPD demonstrate that repeated MDRT sessions can improve FEV_1_%, inspiratory capacity, maximal inspiratory and expiratory pressures, diaphragmatic excursion, and functional exercise capacity [9].

However, the relevance of these findings to mechanically ventilated neurocritical patients remains unknown. Unlike COPD or non-neurocritical ICU populations, neurocritical patients face a compounded burden: prolonged mechanical ventilation, markedly reduced spontaneous diaphragmatic activity, sedation exposure, and disrupted neural–respiratory coupling. These factors position them at a unique intersection of muscular and neurological vulnerability, potentially exacerbating VIDD and influencing neurological outcomes through altered afferent signaling and systemic inflammatory responses [5,6].

Accordingly, the primary objective of this pilot study was to evaluate the feasibility of implementing MDRT in neurocritical patients undergoing invasive mechanical ventilation and to obtain preliminary estimates of its immediate effects on diaphragmatic kinematics (secondary outcome) to inform the development of a future clinical trial.

## 2. Materials and Methods

This was a randomized, parallel-group pilot trial with allocation concealment and blinded outcome assessment. It was conducted in the Intensive Care Unit (ICU) of a public hospital in northeast Brazil. The protocol was approved by the Ethics and Research Commission (CEP) of the University of Pernambuco—CAAE 29528519.1.0000.5191 (Approval Date: 30 March 2020), and the trial registered in the Brazilian Registry of Clinical Trials (ReBEC) under the identification: RBR-3ngffwr. The study followed CONSORT 2025 guidelines, including the extension for pilot and feasibility trials [10].

As participants exhibited reduced levels of consciousness, their legal representatives were thoroughly informed about the study’s objectives, procedures, and potential risks, and provided verbal authorization followed by written informed consent prior to enrollment. If they agreed to participate, they signed the Informed Consent Form in accordance with resolution 466/2012. The Informed Consent Form was obtained after a thorough explanation of the study objectives, procedures, risks, and benefits, allowing the participants to make an informed decision about their participation.

### 2.1. Population

The study population consisted of neurocritical care patients who were admitted to the unit and underwent IMV for more than 24 h. Due to accessibility, the sample was non-probabilistic and selected by convenience. Neurocritical patients are the intended clinical population for whom MDRT would be applied in future efficacy trials. Due to prolonged sedation, impaired central respiratory drive, and altered neural–respiratory coupling, these patients are at particularly high risk of VIDD. Therefore, they are the most relevant group to initially test the feasibility and safety of MDRT. Studying healthy volunteers would not capture the technical and safety challenges specific to delivering MDRT to sedated or aroused, mechanically ventilated neurocritical patients, such as ventilator synchrony and the need for continuous hemodynamic monitoring.

At the time of data collection, the unit followed a light-sedation protocol with daily sedation interruption. Therefore, both diaphragm ultrasound assessments and MDRT sessions were performed during these scheduled interruptions when the patients were off continuous sedative infusions, awake or arousable, and clinically stable under pressure-support ventilation (PSV) mode. This approach ensured patient safety and physiological responsiveness during the intervention period.

**Figure 1:**
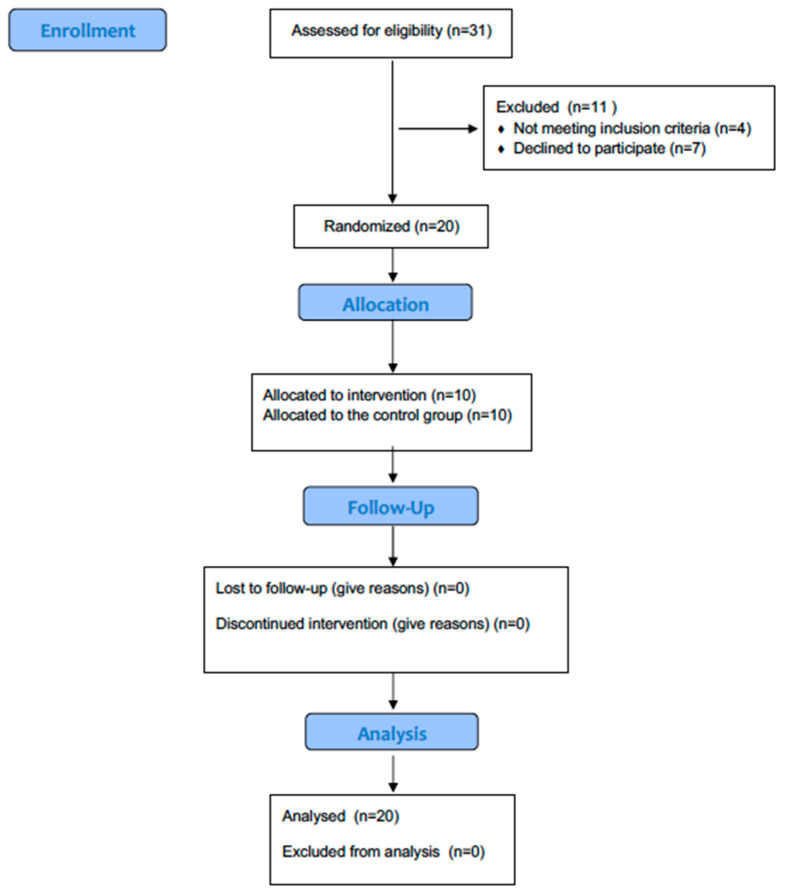
CONSORT flow diagram detailing the number of participants screened, enrolled, allocated to each group, assessed for outcomes, and included in the final analysis.

Randomization was performed at a 1:1 ratio using permuted blocks of four. An independent statistician generated the random sequence using Random.org. This statistician had no role in screening, recruitment, or outcome assessment. The independent statistician implemented allocation with sequentially numbered, opaque, sealed envelopes. The ICU research coordinator stored the envelopes and opened them only after a participant had been enrolled. The person who opened the envelope (the ICU research coordinator) was not involved in outcome assessment. Outcome assessors remained blinded to group allocation throughout data collection. The flow of participants throughout the study is presented in Figure 1.

Notably, this pilot study was designed as an immediate-effect evaluation in a single session for three pragmatic reasons. First, the primary objectives were feasibility and immediate tolerability in the target clinical population. This information is best captured in a short, well-monitored procedure before attempting any longer exposure. Second, safety signals and procedural logistics, such as patient positioning, ultrasound acquisition under PSV, and interference with ventilator waveforms, are most efficiently and ethically established via an acute assessment in high-risk patients rather than in healthy volunteers. Third, single-session data provide preliminary variance estimates for ultrasound kinematic measures necessary to adequately plan powered, multi-session efficacy trials. A single-session evaluation does not preclude later multi-session studies. Rather, it is the first required step in a staged, risk-minimizing translational approach.

### 2.2. Eligibility Criteria

Patients were eligible for inclusion if they were 18 years of age or older, of either sex, and admitted to the neurocritical care unit with a diagnosis of primary neurological disorders, such as stroke, traumatic brain injury, or hypoxic–ischemic encephalopathy. Additional inclusion criteria were: IMV for more than 24 h, presence of an endotracheal tube, and being ventilated in PSV with positive end-expiratory pressure (PEEP) < 8 cmH_2_O at the time of enrollment.

Exclusion criteria included tracheostomy, hemodynamic instability, uncontrolled intracranial hypertension, acute neurological deterioration, hypotension (mean arterial pressure < 60 mmHg), use of neuromuscular blocking agents, or requirement for FiO_2_ > 60% or PEEP > 8 cmH_2_O. Patients were also excluded if they were undergoing continuous renal replacement therapy, exhibited ventilatory discomfort (respiratory rate > 30 breaths/min, SpO_2_ < 90%, or use of accessory respiratory muscles), had body mass index (BMI) > 35 kg/m^2^, a history of recent thoracic or abdominal surgery, or the presence of a chest tube.

Furthermore, any clinical condition preventing safe manual diaphragm mobilization was considered an exclusion criterion. All included patients were hemodynamically stable, clinically monitored, and free from contraindications to chest physiotherapy or manual thoracic manipulation.

### 2.3. Baseline Assessment and Outcome Measures

Anthropometric, clinical and demographic data were collected immediately before the intervention to compose the baseline of the studied population. The participants were evaluated at two moments: before performing the technique and immediately after the intervention. This assessment was performed by an examiner who did not know the volunteers’ allocation group.

The primary outcome was feasibility, which was defined as the recruitment rate, adherence to the intervention protocol, and the incidence of intervention-related adverse events. Adverse events were defined as the occurrence of any of the following during or immediately after the intervention: (1) hemodynamic instability (mean arterial pressure < 60 mmHg or >30% variation from baseline), (2) sustained oxygen desaturation (SpO_2_ < 90% for more than 30 s), (3) respiratory distress (respiratory rate > 30 breaths per minute or use of accessory muscles), (4) new, clinically significant arrhythmia detected via continuous electrocardiogram (ECG), (5) uncontrolled vomiting, and (6) reported or observed pain or distress that prevented continuation of the maneuver. Predefined stopping rules mandated the immediate interruption of the procedure if any of these criteria were met, and all events were documented.

The exploratory secondary outcome was immediate diaphragmatic kinematics, which included diaphragmatic contraction velocity, diaphragmatic relaxation velocity, and diaphragmatic excursion (inspiratory and expiratory). Diaphragm thickness was measured at baseline for sample characterization. Kinematic variables were collected to obtain preliminary variance estimates and inform sample size calculations for a definitive trial.

A single physiotherapist with over eight years of respiratory experience performed all ultrasound acquisitions and measurements. This physiotherapist completed a standardized training log of over 70 supervised diaphragmatic ultrasound examinations prior to study initiation. While we did not conduct a formal intra-rater reliability study in this trial, the operator adhered to a standardized measurement protocol (M-mode landmarking and averaging of five consecutive measurements) to minimize measurement error. Future studies will include formal intra- and inter-rater reliability assessments.

### 2.4. Experimental Protocol

#### 2.4.1. Diaphragm Thickness Assessment

The diaphragm thickness assessment was performed using ultrasound (LOGIQ E, General Electric Healthcare, Chicago, IL, USA) in B mode. The patient was positioned in the supine position at 45° and a high-resolution, low-penetration linear transducer (7 MHz) was placed at right angles to the rib cage between the eighth and ninth intercostal space on the right, laterally between the anterior and medial axillary lines [9]. Diaphragm thickness was evaluated at functional residual capacity, when the diaphragm is in a relaxed position. Patients were observed continuously throughout the respiratory cycle, and image acquisition was standardized by freezing the screen at the end of passive expiration, which corresponded to the end-expiratory phase of tidal breathing. This procedure minimized variability in measurements associated with ventilator-patient interaction and ensured a consistent assessment across all participants. Ultrasound assessments were performed under each patient’s clinically indicated pressure-support level, which was kept constant between pre- and post-measures for safety.

#### 2.4.2. Diaphragmatic Excursion and Contraction/Relaxation Velocities Assessment

Diaphragmatic excursion was assessed by measuring the craniocaudal displacement of the diaphragmatic dome along the vertical axis during a full respiratory cycle, from the peak of inspiration to the end of complete expiration. All measurements were performed with patients in a 45° semi-recumbent position to ensure an optimal acoustic window and reproducibility across assessments. Measurements were performed using M-mode ultrasound with a convex transducer (4 MHz) positioned in the mid-axillary line, during spontaneous breathing efforts under pressure support ventilation to ensure consistent patient participation across measurements.

Next, the vertical distance between the point corresponding to the start of full inspiration (Point P1) and the point corresponding to maximum diaphragm displacement (Point P2) was determined for each image recorded in M mode. Diaphragm velocity was determined from diaphragmatic contraction measurements as the ratio between inspiratory contraction amplitude and inspiratory time. Five consecutive measurements were acquired for all variables, obtaining the average of these.

#### 2.4.3. Intervention

**Figure 2:**
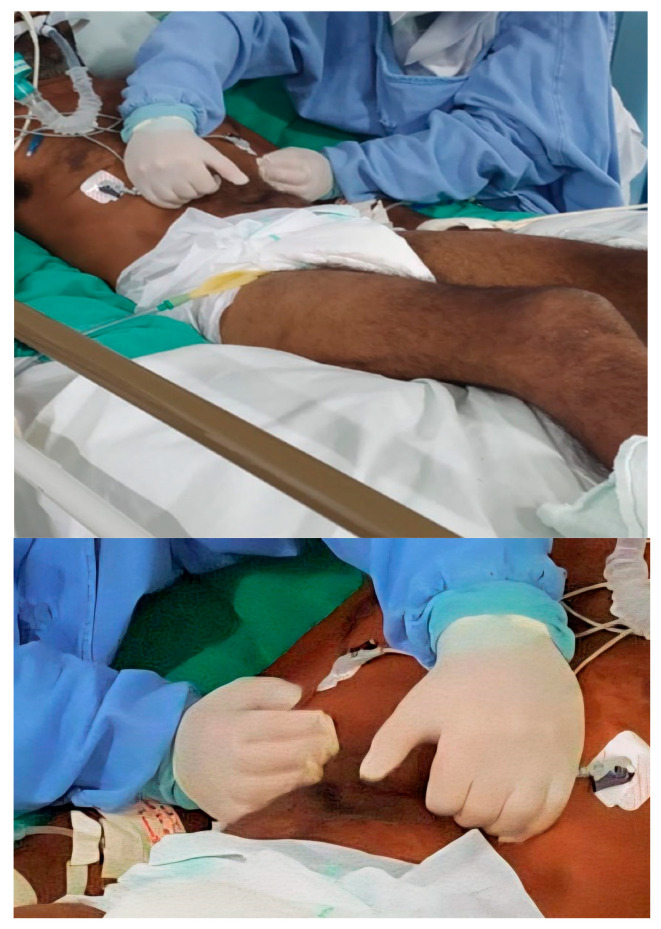
Manual Diaphragm Release Technique (MDRT) applied with the patient in the supine position.

The intervention group (IG) received conventional physiotherapy, which included passive mobilizations, pulmonary secretion clearance, passive cycle ergometry, and ventilatory assistance as needed, combined with the MDRT. Participants were positioned supine at a 45° angle. The therapist stood at the head of the bed and placed both hands along the lower costal margin (ribs 7–10). During inspiration, the therapist applied gentle cranial traction following the physiologic elevation of the ribs. During expiration, the therapist maintained a sustained myofascial hold in a medial and cranial direction. Contact depth was gradually increased across respiratory cycles. The technique consisted of two sets of ten deep breaths, with a one-minute rest between sets (Figure 2). The rationale is that targeted myofascial release reduces restriction at the diaphragm’s insertions, enhances tissue compliance and excursion, and transiently modulates length-tension relationships. These relationships were evaluated through immediate kinematic responses.

The control group (CG) received conventional physiotherapy and a simulated sham maneuver. For this procedure, the therapist positioned their hands identically to the MDRT procedure, but without applying therapeutic pressure, traction, or diaphragmatic mobilization. This controlled for tactile input and therapist presence, while avoiding physiological effects.

Since the ICU had adopted a daily awakening protocol, none of the patients were under continuous sedation during data collection. This ensured that assessments were performed during spontaneous consciousness and ventilatory stability.

Throughout the intervention, patients were monitored for potential discomfort or adverse responses. No session required interruption. According to the study protocol, the maneuver would have been discontinued if clinically significant changes were observed, such as oxygen saturation below 90%, vomiting, pallor, intense sweating, hypotension, arrhythmias on an electrocardiogram, or an increase in heart rate exceeding 20 beats per minute above resting values. Standardized positioning, timing, and manual contact were adopted to minimize allocation bias, as full blinding is not feasible in manual therapy trials.

#### 2.4.4. Data Analysis

As a pilot feasibility trial, our primary objective was to evaluate feasibility, rather than test efficacy. In line with the methodological guidance for pilot trials, we selected a sample size that falls within the commonly recommended range of 12–30 participants to provide sufficient precision to estimate feasibility parameters and variance for continuous outcomes [11]. Therefore, we chose a total sample size of 20 participants (10 per group) to balance practical constraints with the need to obtain preliminary variance estimates for diaphragmatic kinematics.

The Shapiro–Wilk and Levene’s tests were used to assess normality and homogeneity of variance. Continuous parametric data were reported as mean ± standard deviation (SD), while non-parametric variables (e.g., APACHE II, SOFA, GCS) were reported as median [Q1, Q3]. Categorical variables (e.g., sex) were expressed as counts and percentages and analyzed using the Chi-square test of independence.

Between-group comparisons were conducted using independent samples t-tests for parametric data, and Mann–Whitney U tests for non-parametric data. Within-group comparisons used paired t-tests or Wilcoxon signed-rank tests as appropriate. Bonferroni correction was applied for multiple comparisons. Effect sizes (Cohen’s d) were calculated using G*Power version 3.1.7 (Franz Faul, Universität Kiel, Kiel, Germany). A significance level of *p* < 0.05 was considered for all tests. Analyses were conducted using SPSS version 21.0 (SPSS Inc., Chicago, IL, USA).

## 3. Results

A total of 20 patients were included in the study (CG = 10; IG = 10), with no losses recorded. Table 1 presents the baseline characteristics of the participants; although no clinically relevant imbalances were identified, small numerical differences were expected given the pilot nature of the sample.

### 3.1. Feasibility Outcomes

During the recruitment period (May 2021 to January 2023), 40 patients were screened for eligibility. Of these, 25 met the inclusion criteria, and 20 were successfully enrolled and randomized (recruitment rate: 80%). All 20 participants completed the single intervention session as per protocol (protocol adherence: 100%). The mean (±SD) duration for the application of the MDRT or sham maneuver was 6.2 ± 1.1 min. No adverse events were observed in either group during or after the intervention.

### 3.2. Efficacy Outcomes

The analysis of preliminary efficacy endpoints, adjusted for baseline values, showed no statistically significant differences between the Intervention Group and the Control Group immediately after the single session. For the primary efficacy endpoint (diaphragmatic velocities), the adjusted mean difference for contraction velocity was 0.1 mm/s (95% CI −0.3 to 0.5, *p* = 0.50), and for relaxation velocity, it was 0.2 mm/s (95% CI −0.05 to 0.45, *p* = 0.11). Regarding the secondary efficacy endpoints (diaphragmatic excursion), the adjusted mean difference for inspiratory excursion was 0.5 mm (95% CI −1.2 to 2.2, *p* = 0.55), and for expiratory excursion, it was 1.0 mm (95% CI −0.1 to 2.1, *p* = 0.08). Detailed results are presented in Table 2.

To further characterize the precision of the exploratory physiological estimates, we calculated the widths of the 95% confidence intervals for each adjusted between-group difference. The CI widths were 0.8 mm/s for diaphragmatic contraction velocity, 0.5 mm/s for relaxation velocity, 3.4 mm for inspiratory excursion, and 2.2 mm for expiratory excursion. These intervals are consistent with the wide dispersion typically observed in feasibility samples and suggest that large, immediate effects of a single MDRT session are unlikely. Nevertheless, they remain compatible with small-to-moderate acute effects that could accumulate across repeated sessions and become clinically meaningful. Accordingly, these precision estimates provide the variance structure and plausible effect-size range necessary to inform sample-size calculations for a future definitive randomized controlled trial.

Table 3 presents the mean values of the parameters from before and after the intervention for the two groups.

## 4. Discussion

This pilot randomized trial demonstrates that MDRT is both feasible and well tolerated in mechanically ventilated neurocritical patients. We achieved an 80% recruitment rate among eligible participants, full protocol adherence, and a brief intervention duration (6.2 ± 1.1 min). No predefined safety events occurred, supporting the tolerability of the procedure in this high-risk population. Establishing feasibility and safety in the intended clinical setting is a necessary foundation for subsequent efficacy trials; thus, our analysis of immediate diaphragmatic responses should be interpreted as exploratory rather than definitive.

The larger descriptive increases in excursion observed in the control group likely reflect natural recovery during ventilator weaning and breath-to-breath variability under pressure-support ventilation, rather than a true opposite effect. This pattern aligns with the wide confidence intervals and the absence of significant adjusted between-group differences, suggesting that these fluctuations represent expected variability in a small pilot sample. Excursion is influenced by pressure-support level. Although the PS range used reflects routine practice in neurocritical care, higher support may reduce sensitivity of ultrasound to detect intrinsic contractility. Future trials should standardize ventilator settings during assessments. Because the control group had slightly higher pressure-support levels, part of the difference in excursion may be attributable to ventilatory assistance rather than physiological change, reinforcing the need for standardized PS in future studies

Given the clinical relevance of mitigating diaphragmatic inactivity as a driver of VIDD, we evaluated the acute effects of a single MDRT session on diaphragmatic kinematics. No detectable immediate changes were observed in excursion or contraction–relaxation kinetics compared with standard care. Because this was a feasibility trial, the absence of statistically significant between-group differences should not be interpreted as evidence of inefficacy; instead, it reflects the limited statistical precision inherent to small pilot samples. Consistent with CONSORT guidance [10], the exploratory physiological analyses were intended to estimate variance and plausible effect-size ranges rather than to support hypothesis testing. In this context, the 95% confidence intervals observed here are informative: their breadth captures the uncertainty typical of feasibility designs, while their limits help delineate the range of short-term effects that a fully powered trial should be designed to detect. Accordingly, the terminology used emphasizes that no detectable differences were observed, which is consistent with the exploratory nature of a feasibility trial not powered to assess efficacy.

To our knowledge, this is the first study to examine MDRT in neurocritical patients, a population in whom IMV-related constraints limit both the magnitude of expected physiological responses and the statistical power to detect them. Accordingly, these findings should be viewed as groundwork rather than evidence of therapeutic benefit. While MDRT appears feasible and safe in this context, its routine use should await confirmation from larger, well-designed trials.

Ultrasound-derived metrics such as diaphragmatic excursion and contraction velocity correlate with clinically relevant outcomes including successful weaning, extubation, and ICU survival [12]. Although not validated surrogate endpoints, these measures provide early biological signals and critical variance estimates that strengthen the design of future clinical trials. To aid interpretation of the numerical patterns observed such as the nonsignificant *p* = 0.08 for expiratory excursion it is important to contextualize these values within the framework of clinical meaningfulness. At present, no validated minimal clinically important difference (MCID) exists for ultrasound-derived diaphragmatic excursion or velocity in mechanically ventilated neurocritical patients. These metrics are typically used as predictive markers (e.g., for extubation success) rather than outcomes with established patient-centered thresholds. Accordingly, the observed changes cannot be classified as clinically meaningful or negligible based on MCID criteria. The borderline *p*-value should therefore not be interpreted as a trend or early signal of benefit; rather, it reflects imprecision and overlap with normal biological variability under pressure-support ventilation. Its primary utility lies in helping to bound the range of plausible effect sizes that should guide sample-size calculations in a future multi-session RCT.

In our cohort, diaphragm thickness values were within the expected physiological range: 1.8 ± 0.4 mm in the intervention group and 1.9 ± 0.5 mm in the control group at baseline. These findings align with reported reference values for healthy adults, which range from 1.5 to 2.0 mm [12] and 1.8 to 3.0 mm [13]. Nevertheless, diaphragm thickness assessment is technically challenging and subject to variability due to factors such as probe frequency, difficulty performing measurements in obese patients, the 0.1 mm resolution limit of ultrasound devices, and inter-operator measurement variability [14]. In addition, no difference after the intervention in this study was observed in inspiratory and expiratory excursion. However, the mean pre-intervention inspiratory excursion values were between 20.85 mm ± 5.3 in the IG and 19.93 mm ± 7.1 in the CG. The cut-off point of diaphragm excursion for diaphragmatic dysfunction is between 10 and 14 mm in spontaneous breathing [13], but there is no previous data available on reference values for patients on IMV, even in pressure support ventilation mode. The assessment of diaphragm excursion is a method which has high sensitivity (93%) and specificity (100%) for detecting diaphragm dysfunction [14], so it is believed that the absence of previous diaphragm dysfunction may have been an influencing factor in the findings observed in this study.

This study only evaluated the acute effects of one MDRT session, and the cumulative effects of the intervention on the outcomes studied were not evaluated. In a randomized clinical trial, Rocha et al. [9] evaluated 19 older adults with COPD who received MDRT in non-consecutive sessions and found that the intervention increased diaphragm excursion in this population, but these results were only achieved after 6 treatment sessions, with a difference between the cumulative 18 mm groups (95%CI 8 to 28). Future investigations should consider a randomized crossover design to control for inter-patient variability, with repeated measures over multiple sessions. This approach would help disentangle true intervention effects from natural temporal improvements and further test the cumulative efficacy of MDRT.

One of the objectives of MDRT is to stretch diaphragm fibers to enhance overall diaphragmatic excursion. However, since the diaphragm is primarily an inspiratory muscle [15] and passive expiration predominates under mechanical ventilation, particularly in pressure support mode, improvements in expiratory excursion are not necessarily expected or clinically meaningful in this context.

The absence of MDRT effect on diaphragm extensibility may be more accurately explained by the underlying mechanisms of VIDD, which include compromised perfusion, contractile dysfunction, and muscle fatigue rather than reduced range of motion per se. PEEP-induced shortening of diaphragm fibers may limit mechanical efficiency but is unlikely to be reversed with acute stretching. Animal studies have demonstrated that alterations in endothelial function and vascular resistance in the diaphragm substantially impair perfusion [5,6,16] suggesting that interventions targeting vascular mechanisms may be more relevant than passive stretching. The velocity of diaphragmatic contraction is considered an indirect measure of diaphragm contraction force. A value of 29 mm/s was reported by Varón-veja et al. [17] as a predictive value for successful extubation, with 13 mm/s being considered a normal value. The velocity of diaphragmatic contraction after the intervention in this study did not change, as indicated by the ANCOVA results adjusted for baseline (adjusted mean difference 0.1 mm/s, 95% CI −0.3 to 0.5, *p* = 0.50). However, it was also observed that the evaluated sample had velocity of diaphragmatic contraction values within the normal range for the healthy population at baseline (IG = 19.5 ± 5.5 and CG = 19.69 ± 7.0), which may justify our results.

A study seeking to verify the effect of manual therapy on the diaphragm [18] evaluated spontaneous breathing in 40 sedentary women aged between 18 and 35 years. The authors observed that the MDRT influences respiratory muscle strength due to the increase in maximum expiratory pressure and excursion of the thoracic cavity. However, this study used a combination of techniques, which differentiates its intervention protocol from that proposed in our study. Therefore, we again suggest that the use of IMV with PEEP may limit the effects of the MDRT in this population. Therefore, the variables that directly depended on the range of movement and muscle contractile capacity may have suffered from limitations in the effectiveness of the technique, especially since they only evaluated the acute effect after a single session.

The present study is not without its limitations. Firstly, given the experimental nature of this pilot study, it is not possible to ascertain with certainty that MDRT exerts no effect on diaphragmatic kinematics. Secondly, the inability to observe the cumulative effects of the technique may have constrained its effect size. Third, all ultrasound assessments were performed by a single operator; although this minimized inter-operator variability, it precludes assessment of inter-rater reliability. However, this study is pioneering in its examination of the effects of MDRT in individuals undergoing invasive mechanical ventilation, and its results may support future hypotheses regarding the application of this technique as a proposal to facilitate ventilatory weaning and rehabilitation of this population. Furthermore, the study had the additional limitation of only one physiotherapist being evaluated.

Despite the absence of substantial findings regarding immediate kinematic changes following a solitary session, the demonstration of feasibility constitutes a pivotal inaugural step. The significance of recognizing and treating diaphragm dysfunction in critically ill patients, particularly those in the neurocritical population who are susceptible to prolonged ventilation, underscores the necessity for further investigation into interventions such as MDRT. Future studies should incorporate the lessons learned from this pilot, employing larger sample sizes calculated based on these preliminary data (or relevant literature) and potentially exploring the effects of multiple MDRT sessions over a longer duration. In addition, the interpretability of nonsignificant findings such as the *p* = 0.08 observed for expiratory excursion should be understood in the context of feasibility trial methodology. Because the study was not powered for hypothesis testing, such values primarily reflect imprecision rather than absence of effect. The wide confidence intervals observed here are expected in early-phase studies and are valuable for bounding plausible effect sizes and informing sample-size calculations for a fully powered future RCT.

## 5. Conclusions

This pilot randomized controlled trial demonstrated that the Manual Diaphragm Release Technique is a feasible and well-tolerated intervention for neurocritical patients undergoing invasive mechanical ventilation. Although no immediate effects on diaphragmatic kinematics were detected after a single session, the absence of adverse events, high protocol adherence, and full recruitment compliance provide strong evidence supporting its clinical applicability and methodological feasibility in this setting.

These findings establish a solid foundation for future research aimed at exploring the physiological and clinical effects of repeated MDRT sessions. Larger, adequately powered multicenter trials with extended follow-up periods are warranted to determine whether cumulative application of MDRT can favorably influence diaphragmatic function, ventilator weaning, and long-term respiratory outcomes. By confirming feasibility in a highly vulnerable population, this study contributes an important step toward integrating manual diaphragm release into early, multidisciplinary strategies for respiratory muscle preservation and rehabilitation in critical care.

## Figures and Tables

**Table 1:** Baseline characteristics of participants.

Variable	IG (*n* = 10)	CG (*n* = 10)	*p*-Value
Age (years)	55.1 ± 16.9	58.3 ± 15.2	0.635
Sex (male/female)	6/4	7/3	0.655
BMI (kg/m^2^)	25.9 ± 3.5	26.8 ± 4.1	0.571
APACHE II score	18.5 ± 3.3	19.2 ± 5.2	0.789
SOFA score	7.1 ± 3.0	7.8 ± 3.0	0.592
Glasgow Coma Scale	8 ± 1.5	8 ± 3.0	0.841
Days of IMV	5.3 ± 3.1	6.1 ± 3.8	0.567
PS (cmH_2_O)	9.8 ± 3.15	12 ± 5.7	0.186
PEEP (cmH_2_O)	5.7 ± 1	5.5 ± 0.9	1.000
Cause of ICU admission			0.655
Stroke	5 (50%)	6 (60%)	
Traumatic brain injury	3 (30%)	2 (20%)	
Hypoxic–ischemic injury	2 (20%)	2 (20%)	
Diaphragm thickness (mm)	1.8 ± 0.4	1.9 ± 0.5	0.581

Values are presented as mean ± standard deviation or n (%). BMI: Body Mass Index; APACHE II: Acute Physiology and Chronic Health Evaluation II; PS: Pressure Support; PEEP: Positive end-expiratory pressure; SOFA: Sequential Organ Failure Assessment; IMV: Invasive Mechanical Ventilation; IG: Intervention Group; CG: Control Group.

**Table 2:** ANCOVA-Adjusted Effects of the Manual Diaphragm Release Technique (MDRT) on diaphragmatic kinematics.

Variable	Adjusted Mean Difference (IG − CG)	95% Confidence Interval	*p*-Value
Contraction Velocity (mm/s)	0.1	−0.3 to 0.5	0.50
Relaxation Velocity (mm/s)	0.2	−0.05 to 0.45	0.11
Inspiratory Excursion (mm)	0.5	−1.2 to 2.2	0.55
Expiratory Excursion (mm)	1.0	−0.1 to 2.1	0.08

Values represent the estimated difference in post-intervention means between the Intervention Group (IG) and Control Group (CG), adjusted for the baseline value of the respective outcome. Positive values indicate a higher mean in the IG. CI: Confidence Interval.

**Table 3:** Mean (SD) values of diaphragmatic contraction velocity, relaxation velocity, and excursion for each study group.

Variable	IG			CG		
	pre	post	SD	pre	post	SD
Contraction velocity (mm/s)	19.5 + 5.5	21.5 + 6.3	2.54	19.69 + 7.0	25.4 + 13.2	5.78
Relaxation Velocity (mm/s)	12.2 + 6.3	12.0 + 5.2	−0.24	14.9 + 9.3	15.9 + 8.9	1.05
Inspiratory Excursion (mm)	20.85 + 5.3	21.6 + 5.1	0.79	19.93 + 7.1	22.3 + 8.5	2.39
Expiratory Excursion (mm)	22.37 + 4.8	22.3 + 3.8	−2.72	21.1 + 7.4	24.8 + 8.6	3.70

## Data Availability

The original contributions presented in this study are included in the article. Further inquiries can be directed to the corresponding author.

## Abbreviations

The following abbreviations are used in this manuscript:

MDRT	Manual Diaphragm Release Technique
IMV	Invasive mechanical ventilation
TBI	Traumatic brain injury
HIBI	Hypoxic–ischemic brain injury
VIDD	Ventilator-induced diaphragmatic dysfunction
PEEP	Positive End-Expiratory Pressure

## References

[1] Borsellino B., Schultz M.J., Abreu M.G., Robba C., Bilotta F. (2016). Mechanical ventilation in neurocritical care patients: A systematic literature review. Expert Rev. Respir. Med..

[2] Grosu H.B., Lee Y.I., Lee J., Eden E., Eikermann M., Rose K. (2012). Diaphragm muscle thinning in patients who are mechanically ventilated. Chest.

[3] Spiesshoefer J., Kersten A., Geppert J.E., Regmi B., Senol M., Kabitz H.J., Dreher M. (2023). State-of-the-art opinion article on ventilator-induced diaphragm dysfunction: Update on diagnosis, clinical course, and future treatment options. Respiration.

[4] Powers S.K. (2024). Ventilator-induced diaphragm dysfunction: Phenomenology and mechanism(s) of pathogenesis. J. Physiol..

[5] Bezerra N.M.S., Oliveira A.L.E.F., Figueirêdo B.B., Schwingel P.A., Magalhães P.A.F. (2025). Diaphragm morphology and function in neurocritical care patients: Uncovering key correlations with respiratory muscle strength under mechanical ventilation. Physiother. Res. Int..

[6] Taran S., Cho S.M., Stevens R.D. (2023). Mechanical ventilation in patients with traumatic brain injury: Is it so different?. Neurocrit. Care.

[7] Zhang J., Feng J., Jia J., Wang X., Zhou J., Liu L. (2023). Research progress on the pathogenesis and treatment of ventilator-induced diaphragm dysfunction. Heliyon.

[8] Bissett B., Gosselink R., van Haren F.M.P. (2020). Respiratory muscle rehabilitation in patients with prolonged mechanical ventilation: A targeted approach. Crit. Care.

[9] Rocha T., Souza H., Brandão D.C., Rattes C., Ribeiro L., Campos S.L., Aliverti A., de Andrade A.D. (2015). The manual diaphragm release technique improves diaphragmatic excursion, inspiratory capacity and exercise capacity in people with chronic obstructive pulmonary disease: A randomised trial. J. Physiother..

[10] Hopewell S., Chan A.-W., Collins G.S., Hróbjartsson A., Moher D., Schulz K.F., Tunn R., Aggarwal R., Berkwits M., Berlin J.A. (2025). CONSORT 2025 statement: Updated guideline for reporting randomized trials. JAMA.

[11] Billingham S., Whitehead A., Julious S. (2013). An audit of sample sizes for pilot and feasibility trials being undertaken in the United Kingdom registered in the United Kingdom Clinical Research Network database. BMC Med. Res. Methodol..

[12] Matamis D., Soilemezi E., Tsagourias M., Akoumianaki E., Dimassi S., Boroli F., Richard J.C., Brochard L. (2013). Sonographic evaluation of the diaphragm in critically ill patients: Technique and clinical applications. Intensive Care Med..

[13] Zambon M., Greco M., Bocchino S., Cabrini L., Beccaria P.F., Zangrillo A. (2017). Assessment of diaphragmatic dysfunction in the critically ill patient with ultrasound: A systematic review. Intensive Care Med..

[14] Boussuges A., Rives S., Finance J., Brégeon F. (2020). Assessment of diaphragmatic function by ultrasonography: Current approach and perspectives. World J. Clin. Cases.

[15] Sieck G.C., Fogarty M.J. (2025). Diaphragm muscle: A pump that cannot fail. Physiol. Rev..

[16] Horn A.G., Davis R.T., Baumfalk D.R., Kunkel O.N., Bruells C.S., McCully D.J., Opoku-Acheampong A.B., Poole D.C., Behnke B.J. (2019). Vasodilation of diaphragm resistance vessels with prolonged mechanical ventilation. J. Appl. Physiol..

[17] Varón-Vega F., Hernández Á., López M., Cáceres E., Giraldo-Cadavid L.F., Uribe-Hernandez A.M., Crevoisier S. (2021). Usefulness of diaphragmatic ultrasound in predicting extubation success. Med. Intensiv. (Engl. Ed.).

[18] Marizeiro D.F., Florêncio A.C.L., Nunes A.C.L., Campos N.G., Lima P.O.P. (2018). Immediate effects of diaphragmatic myofascial release on the physical and functional outcomes in sedentary women: A randomized placebo-controlled trial. J. Bodyw. Mov. Ther..

